# Effectiveness of an Innovative Pulsed Electromagnetic Fields Stimulation in Healing of Untreatable Skin Ulcers in the Frail Elderly: Two Case Reports

**DOI:** 10.1155/2015/576580

**Published:** 2015-11-08

**Authors:** Fabio Guerriero, Emanuele Botarelli, Gianni Mele, Lorenzo Polo, Daniele Zoncu, Paolo Renati, Carmelo Sgarlata, Marco Rollone, Giovannoi Ricevuti, Niccolò Maurizi, Matthew Francis, Mariangela Rondanelli, Simone Perna, Davide Guido, Piero Mannu

**Affiliations:** ^1^Department of Internal Medicine and Medical Therapy, Section of Geriatrics, University of Pavia, 27100 Pavia, Italy; ^2^Agency for Elderly People Services, Hospital Santa Margherita, 27100 Pavia, Italy; ^3^Ambra Elektron, Associazione Italiana di Biofisica per lo Studio dei Campi Elettromagnetici in Medicina, 00186 Rome, Italy; ^4^Alberto Sorti Research Institute, Medicine and Metamolecular Biology, 10122 Turin, Italy; ^5^Department of Public Health, Experimental and Forensic Medicine, Section of Human Nutrition, Endocrinology and Nutrition Unit, University of Pavia, 27100 Pavia, Italy; ^6^Department of Public Health, Experimental and Forensic Medicine, Biostatistics and Clinical Epidemiology Unit, University of Pavia, 27100 Pavia, Italy

## Abstract

*Introduction*. Recalcitrant skin ulcers are a major burden in elderly patients. Specifically, chronic wounds result in significant morbidity and mortality and have a profound economic impact. Pulsed electromagnetic fields (PEMFs) have proved to be a promising therapy for wound healing. Here we describe the first reported case of an innovative PEMF therapy, Emysimmetric Bilateral Stimulation (EBS), used to successfully treat refractory skin ulcers in two elderly and fragile patients.* Case Presentation*. Two elderly patients developed multiple chronic skin ulcerations. Despite appropriate treatment, the ulcers showed little improvement and the risk of amputation was high. Both patients underwent daily EBS therapy and standard dressing. After few weeks of treatment, major improvements were observed and all ulcers had healed.* Conclusion*. In patients with refractory ulceration, EBS therapy may be of real benefit in terms of faster healing. This case supports the supportive role for PEMFs in the treatment of skin ulceration in diabetes and is suggestive of a potential benefit of EBS in this clinical condition.

## 1. Introduction

Due to life expectancy increase and concomitant aging of the population, the prevalence of chronic cutaneous ulcers dramatically increased during the last decades, specifically when arising from atherosclerotic and microangiopathic processes [1, 2].

Several pathophysiological mechanisms related to aging counteract the healing process, such as the physiological loss of trophic dermoepidermal elasticity and concomitant alterations in skin microcirculation. Additionally, the broad spectrum of comorbidities, which often affect the subjects at risk, might further impair the healing process [3].

Wound healing is a complex process mediated by signals of molecular interaction involving the recruitment of mesenchymal cells, proliferation, and regeneration of the extracellular matrix. The healing process is a response of innate immunity for the restoration of tissue integrity. It is regulated by a pattern of events including coagulation, inflammation, granulation tissue formation, epithelialization, and tissue remodeling. These events are mediated by cytokines and growth factors that modulate such cellular activities [4, 5].

Despite the modern advances in wound closure techniques and devices, there is a vital need for newer methods of enhancing the healing process to achieve optimal outcomes.

One of the promising but still debated therapeutic developments involves the use of electromagnetic fields (EMFs) for enhancing the healing process. It is hypothesized that electrical stimulation influences the migratory, proliferative, and synthetic functions of fibroblasts and also results in increased expression of growth factors [6].

This case report shows an innovative technique based on pulsed EMF (PEMF) stimulation, Emysimmetric Bilateral Stimulation (EBS), to enhance the healing of recalcitrant skin ulcers in two fragile elderly patients.

## 2. Cases Presentation

### 2.1. Materials and Methods

EBS therapy was carried out on two elderly patients, whose leg ulcers were chronic and healing was unsatisfactory. Both ulcers had not responded to conventional medication, including washing, disinfection, and advanced dressing for some months before coming to our attention.

We evaluated the effect of this innovative EMF stimulation, using EBS device, by the receding of leg ulcers and secondary biochemical and clinical changes in C-reactive protein (CRP), erythrocytes sedimentation rate (ESR), and comprehensive geriatric assessment (Table 1).

### 2.2. Experimental Protocol

**Figure 1:**
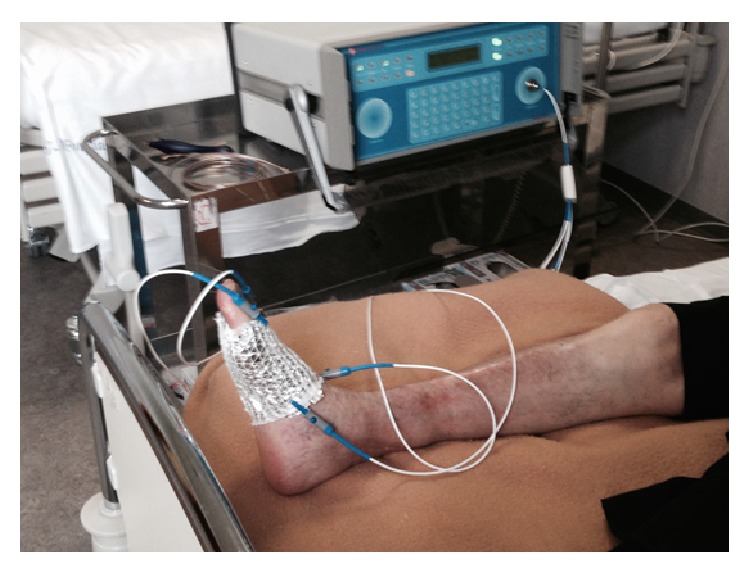
EBS treatment performed at bedside.

Both elderly subjects received EBS stimulation daily after undressing and washing procedure. The intervention was conducted in a supine position with the knee fully extended. The lower leg and foot were supported with a foot stand so the ankle was kept in a neutral ankle position throughout the intervention. Each treatment session lasted for 25 minutes and was repeated daily until the wound healed (Figure 1).

### 2.3. Emysimmetric Bilateral Stimulation (EBS)

Each EBS treatment (Elkmed^©^ 2060) consists of a stimulation (about 20–25 minutes long) during which the patient is exposed to extremely weak EMF (powers in range 10–100 nW), emitted by two sources: an in air-source placed in the front part of the machine and another consisting of a shaped aluminium conductor sheet placed directly on wounds, connected by 4 clamps that convey the signal from the machine. The in air-source at a distance of 1 meter generates electromagnetic power densities in the range of 50–100 nW/cm^2^; local emitters generate even lower power densities, between 0.5 and 50 nW/cm^2^, depending on the terminal emitter used. Electromagnetic signals are pulsed at a frequency variable by preset programs. The carrier wave is peaked at 10.5 GHz. Pulsation consists of an amplitude modulation at total index (*m* = 1) and is square-shaped so that the carrier wave is switched on/off at a very high rate.

The purpose in designing EBS apparatus was to obtain the largest frequency band possible at extremely low powers in the emission. EBS's working principle is to introduce very weak electromagnetic fields noise in the range of radiomicrowave frequencies into the network of molecular chains interpenetrating the whole biological matter. The EMF noise is obtained creating many harmonics and interferences of electromagnetic weak signals within the exposed body. This is the ideal condition in order to obtain extremely low power electromagnetic noise spread over a wide frequency band, able to stimulate self-feeding and reenhance traveling wave-packets, whose role is involved in biocommunication, homeostasis, and regeneration [7–9].

## 3. Case Report 1

The patient, C. C., was a 91-year-old female, suffering from chronic heart failure and osteoarthrosis with subsequent functional impairment. She was a retired teacher and lived alone without any external caregiving support. She came to our department presenting painful skin ulcers of the lower extremities with inflammatory signs and alarming initial gangrene. They arose from accidental wounds 6 months before and increased in size despite wound care performed by the patient herself (washing, disinfection, dressing with topical silver sulfadiazine, etc.). No history of diabetes was reported.

**Figure 2:**
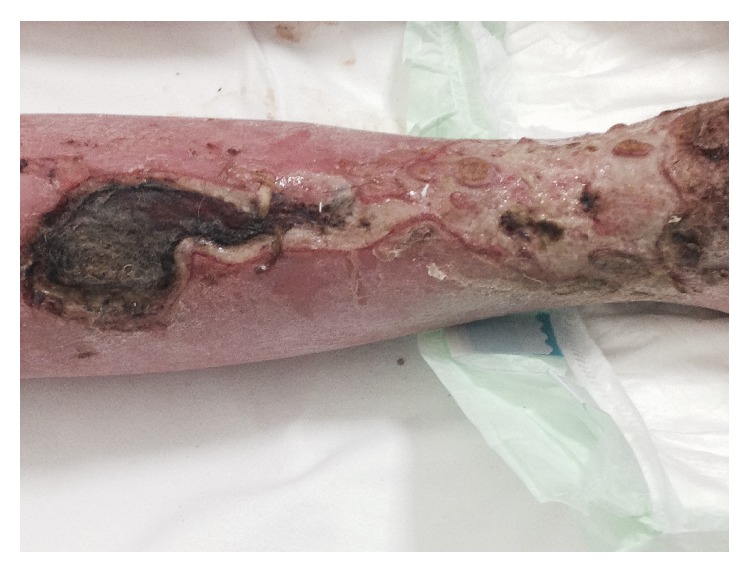
Initial gangrene of the lower limb at admission.

At admission, she presented in poor general condition and was malnourished, while cognitive functions were intact. A skin examination was performed at the time of admission and showed extensive right leg ulcers. These lesions had a necrotic appearance, without granulation tissue, and were covered by purulent exudates (Figure 2).

Blood sample showed increased levels of CRP and ESR. Main clinical data are shown in Table 1. A Doppler ultrasound of the lower limbs showed distally an initial decrease of arterial signal with normal venous axis. The ankle brachial index was 0.9, so that arterial insufficiency was not causing or contributing to the nonhealing wound [10].

Consultation from the vascular surgery department proposed a demolitive surgical approach that was refused. Given the lack of response, risk of amputation, and general deterioration in the patient, EBS stimulation was proposed and started on a compassionate use basis, with informed consent, 2 days after her hospitalization. She parallel underwent standard medication with washing, enzymatic debridement, dressing, and parenteral antibiotics (amoxicillin/clavulanic acid 3 grams daily).

**Figure 3:**
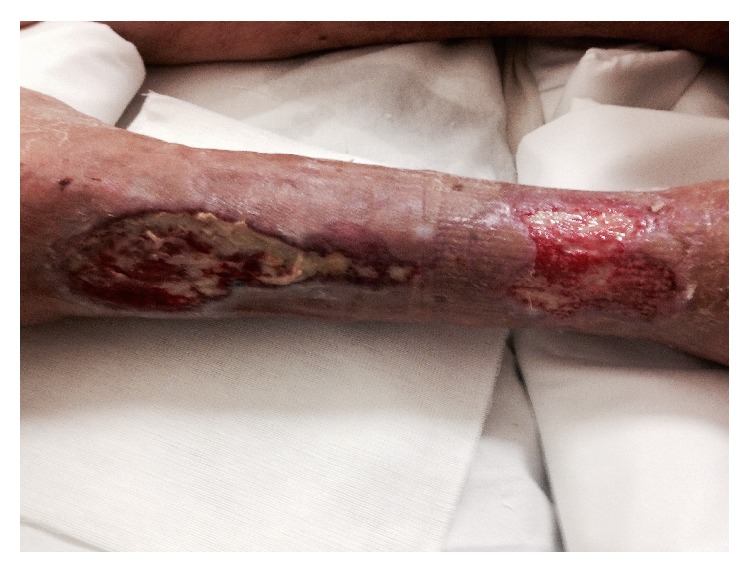
Seven days after EBS treatment and standard dressing.

**Figure 4:**
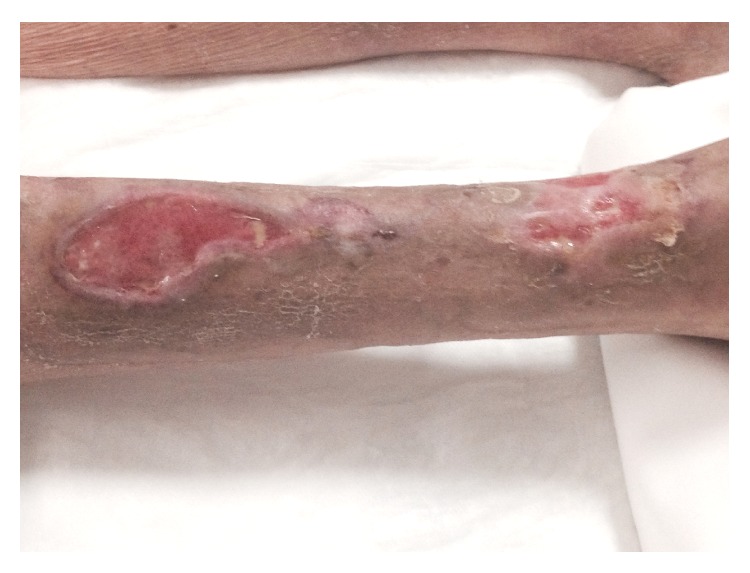
After 2 weeks of EBS treatment and standard dressing.

After 7 days of therapy, the wide ulcers of the lower limb started to present some granulation tissue with irregular edges and discrete fibrinous exudate (Figure 3). Following a two-week EBS treatment period, all wide ulcers of the lower limb had improved with remarkable reduction and marked granulation tissue was apparent on the wide ulcers (Figure 4). The patient's general condition improved in parallel.

**Figure 5:**
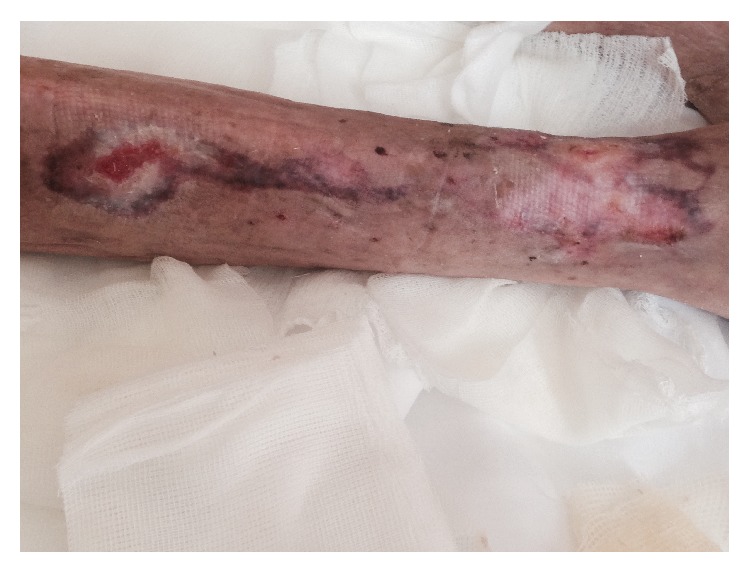
Skin ulcers completely heal after 5 weeks.

Following 5 weeks of EBS treatment, the ulcers on the leg had completely healed (Figure 5) and the patient was able to walk using a walking aid. ESR and CRP consequently decreased to almost normal values.

At discharge ulcer healing was complete with no relapse observed up to now.

## 4. Case Report 2

An 83-year-old diabetic male patient, S. I., was admitted to our department as he was suffering from chronic foot diabetic ulcer that was present for 8 months. Concurrent comorbidities were chronic heart failure, chronic atrial fibrillation, and hyperuricemia.

Until admission, the old man had been for long an outpatient in a diabetic foot center. During this period, a 1 cm ulcer appeared on his right foot after a prolonged walk, and it increased in size despite appropriate wound care and glycemic control. As the ulcer was recalcitrant, tissue drainage surgery had been proposed.

On admission the patient presented in good clinical conditions. The glycemic control was stable with basal bolus scheme adopting rapid short-acting insulin analogue glulisine and long-acting glargine insulin. Glycosylated hemoglobin levels were 51 mmol/mol, and adjustments of his insulin dose and addition of on-demand fast-acting insulin were not required. The patient was treated with oral antibiotics (amoxicillin/clavulanic acid 2 grams/daily) from two weeks before admission. Clinical and biochemical data are shown in Table 1. Wound-related pain represented a relevant issue, as the consequent limitations in mobility with gradual immobilization syndrome.

**Figure 6:**
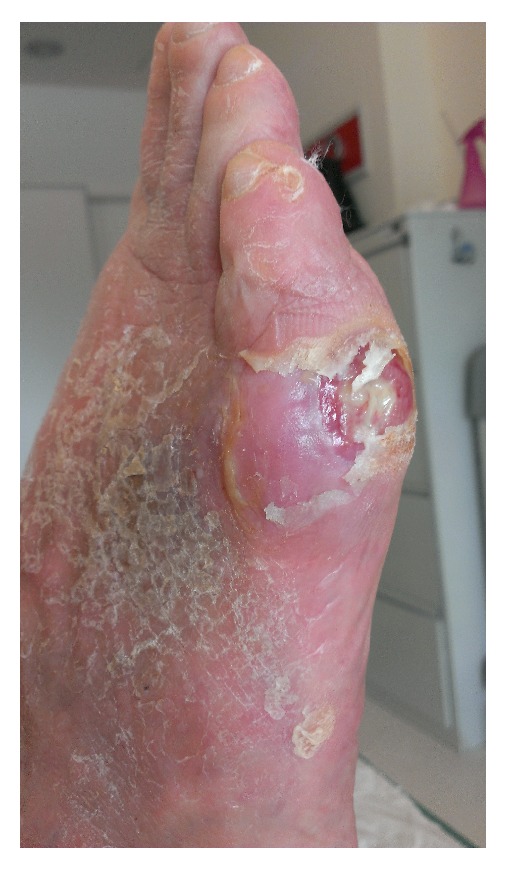
Foot diabetic ulcer at admission.

A skin examination performed at the time of admission showed a foot ulcer of about 2.5 cm involving the fascia, and it was evaluated as grade II according to the Wagner grading system (Figure 6) [11]. An X-ray of the foot was performed and showed no occurrence of osteomyelitis.

Given unresponsiveness to conventional dressings until then and patient's awareness of the longtime expected healing after drainage surgery, EBS therapy was initiated with informed consent. Standard medication with washing, disinfection, l-lysine hyaluronate, and dressing was performed, while antibiotic therapy was administered endovenously.

Since the beginning of EBS stimulation, there was a noticeable improvement of the recalcitrant foot lesion, observed by the increase of granulation tissue, and wound borders receding corresponding to reepithelialization.

**Figure 7:**
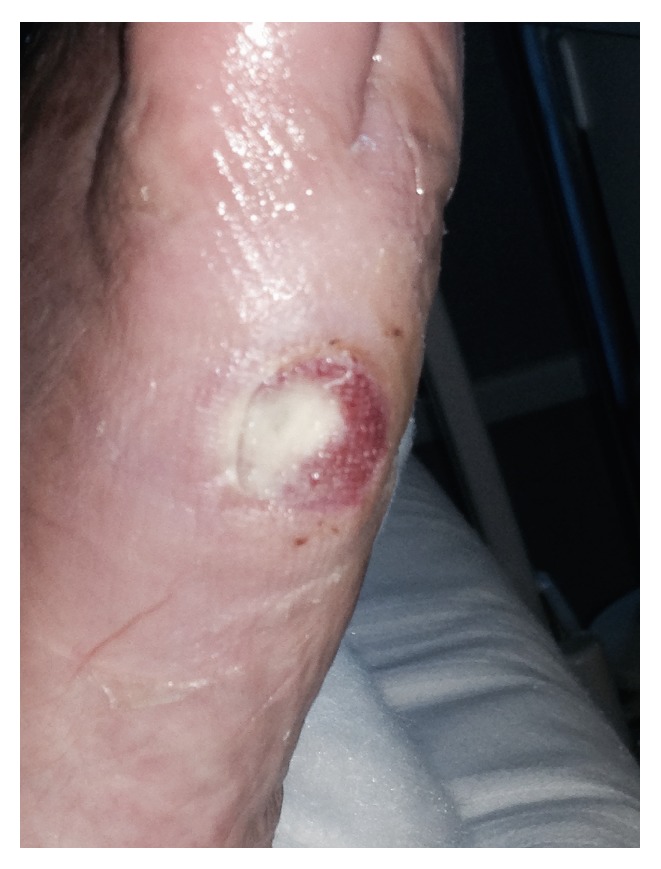
Wound epithelization after 10 days of EBS treatment.

Figure 7 shows changes in the diabetic ulcer, respectively, after 10 days of treatment. As the foot ulcer clinically improved, antibiotics were discontinued, and pain became completely tolerated, making walking possible without any aid.

**Figure 8:**
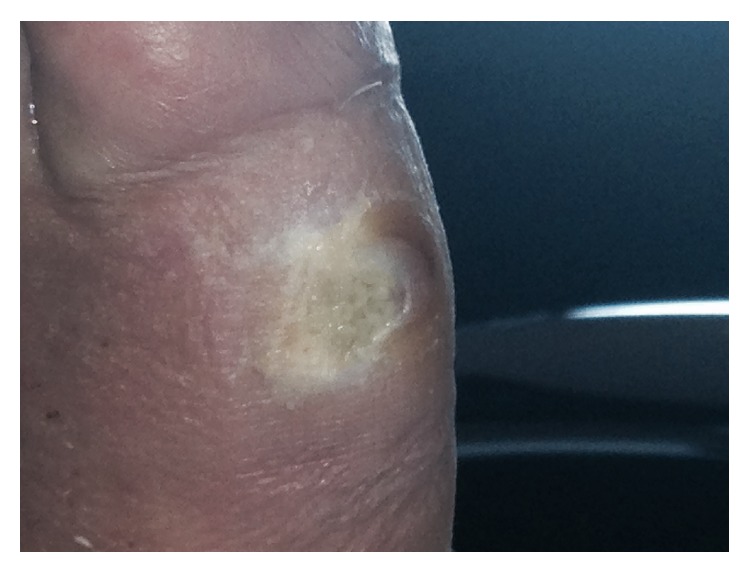
Complete diabetic ulcer healing at discharge.

After 3 weeks of EMF stimulation the ulcer completely healed (Figure 8) and the patient was discharged to home.

## 5. Discussion

In spite of the advancements in cutaneous ulceration treatment, this common condition continues to devastate the community of patients, especially the elderly, who suffer from micro- and macrovascular afflictions. In this respect, the development of new techniques aimed to assist the process of skin healing and repair is of primary importance.

In the last two decades there has been an increasing interest in the PEMF for the management of ischemic, pressure, and venous ulcers. The basic mechanisms underlying EMF are not clear. PEMFs are low frequency fields with very specific shape and amplitude. They can be applied in the presence of a cast or wound dressing and the risk of infection is significantly low [12].

It has been suggested that PEMFs, by altering or augmenting preexisting endogenous electrical fields, may trigger specific, measurable cellular responses such as DNA synthesis, transcription, and protein synthesis [13]. Such cellular responses appear to occur within a window of PEMF parameters (frequency, amplitude, timing, and length of exposures). It has been reported that PEMFs decrease the doubling time of fibroblasts and induce differentiation of skin fibroblasts in culture. Increased collagen synthesis, angiogenesis, and bacteriostasis are some mechanism by which PEMFs may contribute to wound healing [14].

Some recent studies have showed that the treatment with PEMFs may result in shorter healing time and limb function recovery, enhancing the quality of life of the patient. In the treatment of pressure ulcers, three controlled clinical trials tried PEMF but findings were controversial [15–17]. A recent randomized controlled study on 13 diabetic patients confirmed the effectiveness of EMF for promoting the healing in terms of enhancing wound closure and facilitating microcirculation [18]. The authors' findings demonstrated that EMF treatment can elicit vasodilation and increase peripheral blood flow. The increase in microcirculation has been already described to inhibit the inflammation and accelerate the cell proliferation [19].

In this regard a previous animal study showed that PEMFs accelerate time to wound closure, granulation, and cell proliferation in diabetic and normal mice, by upregulation of fibroblast growth factor-2 mediated angiogenesis. Besides in this study EMF was showed to prevent tissue necrosis in response to a standardized ischemic insult [20].

According to our case reports, the sequence of clinical events observed in our patients suggests a beneficial role for EBS stimulation, as no other new or relevant therapeutic intervention concomitant to treatment with EBS was initiated. The relevant issue regarding EBS efficacy was that both patients were suffering from chronic recalcitrant ulcers that were not healing under standard medications and had been proposed for demolitive surgery. EBS stimulation was started after consent of the patients for conservative and compassionate use basis.

EBS stands different than the conventional PEMF stimulation devices as it adopts low power stimulations to cover a wide range of frequency bands, shapes, and durations of pulses of the EMF. The core principle is the utilization of PEMF noise-like stimuli to trigger self-arrangements in the living system of treated subjects and improve wound regeneration. Recently, the group led by Montagnier has detected experimentally the presence of electromagnetic signals originating in the water surrounding biomolecules [21]. To us this should be the key-point of EBS stimulation technique: the stimuli involved in the interaction between human body and extremely weak electromagnetic signals are not energetic but potential and phase based actors, able to produce a phase shift in domains of coherent bound-water constituting cells.

However, as single case reports, several limitations warrant acknowledgement. Wound healing is influenced by multiple variables, and it was not possible to strictly control for all potential confounders in this case. There are many possible confounding variables in these reports, mainly regarding the different characteristics of patients and ulcers, thus the difficulty of conducting standardized studies. Actually studies with cell cultures are carrying on to clarify the biological and molecular effects of EBS on wound healing and cellular regeneration.

Based on literature and on our clinical experience, we conclude that the use of PEMFs and EBS in wound control, although recent, constitutes a very promising technique.

Larger studies should be conducted with lengthier follow-up periods and coverage of randomized population.

## 6. Conclusions

While the observations reported in our reports should be interpreted with caution and need to be confirmed in a controlled study, the sequence of events is suggestive of a beneficial role for EBS therapy in chronic skin ulcers healing. These findings are consistent with recent knowledge on the role of EMF in the treatment of wounds. EBS is a novel EMF stimulation, whose innovative working principle is promising for several clinical applications.

## Figures and Tables

**Table 1:** Clinical and biochemical data of the two patients.

	Patient 1		Patient 2	
	At admission	End EBS	At admission	End EBS
HbA1c (mmol/L)	38	41	51	48
RCP (mg/dL)	20.5	2.3	10.3	1.2
ESR (mm/h)	110	35	75	15
MMSE	26/30	28/30	29/30	29/30
Barthel Index	8/100	44/100	25/100	75/100
NRS	8	0	9	2

^*∗*^HbA1c: glycosylated hemoglobin; RCP: C-reactive protein; ESR: erythrocytes sedimentation rate; MMSE: Mini Mental State Examination; NRS: Numeric Rating Scale.
